# An Assessment of the Penile Squamous Cell Carcinoma Surfaceome for Biomarker and Therapeutic Target Discovery

**DOI:** 10.3390/cancers15143636

**Published:** 2023-07-15

**Authors:** George Daniel Grass, Dalia Ercan, Alyssa N. Obermayer, Timothy Shaw, Paul A. Stewart, Jad Chahoud, Jasreman Dhillon, Alex Lopez, Peter A. S. Johnstone, Silvia Regina Rogatto, Philippe E. Spiess, Steven A. Eschrich

**Affiliations:** 1 Department of Radiation Oncology, H. Lee Moffitt Cancer Center and Research Institute, Tampa, FL 33612, USA; 2 Department of Biostatistics and Bioinformatics, H. Lee Moffitt Cancer Center and Research Institute, Tampa, FL 33612, USA; 3 Department of Genitourinary Oncology, H. Lee Moffitt Cancer Center and Research Institute, Tampa, FL 33612, USA; 4 Department of Anatomic Pathology, H. Lee Moffitt Cancer Center and Research Institute, Tampa, FL 33612, USA; 5 Department of Clinical Genetics, University Hospital of Southern Denmark-Vejle, Beriderbakken 4, 7100 Vejle, Denmark

**Keywords:** penile cancer, surfaceome, human papillomavirus, drug discovery

## Abstract

**Simple Summary:**

Penile cancer is considered a rare disease in most developed countries, yet it represents a significant global oncology challenge. Many patients are faced with substantial psychosocial morbidity from diagnosis and treatment, which is further compounded by a lack of access to novel treatment approaches when the disease becomes resistant to upfront therapies. Proteins on the cell surface, or surfaceome, represent an accessible locale of potential biomarkers and therapeutic targets. This study provides the first description of the surfaceome in penile cancer and evaluates if human papillomavirus infection contributes to surfaceome diversity.

**Abstract:**

Penile squamous cell carcinoma (PSCC) is a rare malignancy in most parts of the world and the underlying mechanisms of this disease have not been fully investigated. About 30–50% of cases are associated with high-risk human papillomavirus (HPV) infection, which may have prognostic value. When PSCC becomes resistant to upfront therapies there are limited options, thus further research is needed in this venue. The extracellular domain-facing protein profile on the cell surface (i.e., the surfaceome) is a key area for biomarker and drug target discovery. This research employs computational methods combined with cell line translatomic (n = 5) and RNA-seq transcriptomic data from patient-derived tumors (n = 18) to characterize the PSCC surfaceome, evaluate the composition dependency on HPV infection, and explore the prognostic impact of identified surfaceome candidates. Immunohistochemistry (IHC) was used to validate the localization of select surfaceome markers. This analysis characterized a diverse surfaceome within patient tumors with 25% and 18% of the surfaceome represented by the functional classes of receptors and transporters, respectively. Significant differences in protein classes were noted by HPV status, with the most change being seen in transporter proteins (25%). IHC confirmed the robust surface expression of select surfaceome targets in the top 85% of expression and a superfamily immunoglobulin protein called *BSG*/CD147 was prognostic of survival. This study provides the first description of the PSCC surfaceome and its relation to HPV infection and sets a foundation for novel biomarker and drug target discovery in this rare cancer.

## 1. Introduction

Penile squamous cell carcinoma (PSCC) is a rare cancer in most developed regions of the world and may be associated with significant psychosocial morbidity secondary to diagnosis and treatment [1,2]. Current estimates suggest that 30–50% of cases are associated with high-risk human papillomavirus (HPV) infection [3], which diversifies the underlying oncogenic machinery contributing to carcinogenesis and disease progression. Limited effective treatment options are available when the disease is locally advanced or refractory to surgical resection, chemoradiation, or first-line chemotherapy [4]. Despite this limitation, targeted therapy has not become a mainstay in PSCC given the rarity of the disease, patient exclusion from most clinical trials, and lack of robust disease models to facilitate translational research [5,6].

The tumor cell surface represents a prime locale for cell-based and drug therapies, which is underscored by prior estimates suggesting more than half of the compounds in the DrugBank database target cell surface proteins [7]. The surfaceome catalogue is a composite of plasma membrane proteins with at least one extracellular domain, which includes functional classes (e.g., receptors, transporters, cell-adhesion molecules) that regulate diverse biologic processes via homo-/heterophilic cell–cell interactions or by autonomous mechanisms. Experimental and computational approaches have described the surface localization of numerous proteins, which provides a platform for further investigation of tumor cell surface protein biology [8,9,10,11,12]. Bausch-Fluck et al. previously used mass spectrometry to generate a Cell Surface Protein Atlas (CSPA) in human and mouse cell types [13]. Subsequently, the CSPA served as a validated training set for a machine-learning approach called SURFY, which evaluated 131 domain-specific features to describe the surfaceome in 610 human cancer cell lines; this work suggested that a snapshot of the human surfaceome is represented by at least 2886 proteins [14].

Although several cancer cell lines were assessed in this seminal in silico surfaceome study, PSCC cells were not analyzed. Similarly, others have also profiled the surfaceome from bulk gene expression data from patient tumors to identify potential therapeutic targets [15,16,17]. To our knowledge, an assessment of the PSCC surfaceome has not been conducted; therefore, in this work we set out to describe the surfaceome of PSCC cells in isolation and within the context of patient-derived tumors. Further, we evaluated whether HPV infection influences the PSCC surfaceome and highlighted potential druggable targets.

## 2. Materials and Methods

### 2.1. Datasets

The datasets used in this study are summarized in Supplemental Table S1.

#### 2.1.1. PSCC Cell Line Translatomic Data

Ribosome-bound RNA expression data were previously analyzed and described [18]. Briefly, mRNA was extracted from polysome fractions based on translating ribosomes and profiled using the Clariom D GeneChip (Thermo Fisher Scientific, Waltham, MA, USA). Since the RNA that was profiled was bound to ribosomes, this represents a pool of transcripts that are more likely to result in protein production compared to evaluation of pooled mRNA. Data were normalized using the SST-RMA algorithm from the Transcriptome Analysis Console (Thermo Fisher Scientific, Waltham, MA, USA). Five HPV-negative PSCC cell lines were arrayed in duplicate; expression was combined across duplicates using the mean.

#### 2.1.2. MCC3651 Gene Expression Data

Following institutional review board approval, 18 frozen patient-derived primary PSCCs were identified. A section of each tumor sample previously underwent high-risk HPV assessment by in situ hybridization and/or diagnostic clinical assessment for HPV DNA. All tissues also underwent immunohistochemistry (IHC) p16 staining [19,20]. RNA was extracted from each tumor and sequenced. Raw fastq reads were trimmed (cutadapt) and aligned using STAR v2.7.7 and quantified by RSEM using gene models from GENCODE (v30). RSEM gene-level expression was imported into the R environment with tximport [21] and RNA expression was estimated using vst (variance stabilizing transformation) from DESeq2 [22].

#### 2.1.3. Johnstone Gene Expression Data

Primary PSCCs were arrayed on an Affymetrix HG-U133 Plus 2.0 GeneChip and have been previously described, including the distribution of HPV infection status [23]. Raw data were normalized using RMA [24].

#### 2.1.4. GSE57955 Gene Expression Data

Penile cancer tumors were arrayed on a two-channel Agilent-014850 Whole Human Genome Microarray (4x44k G4112F) (GPL6480). Raw array data were downloaded from GEO on 5 December 2022. The agilp Bioconductor package was used to loess-normalize individual channel data. Array probes were annotated from GEO platform GPL6480. The control channel (normal pool of 5 autopsied glans) was excluded from further analysis of the surfaceome. Patient characteristics from this cohort were previously described [25].

### 2.2. RNA Sequencing

RNA sequencing libraries were prepared using a Universal RNA-Seq Library Preparation Kit with NuQuant, (Tecan US, Inc., Morrisville, NC, USA). Briefly, 100 ng of DNase-treated RNA was used to generate cDNA and a strand-specific library following the manufacturer’s protocol. Library molecules containing ribosomal RNA sequences were depleted using the NuGen AnyDeplete probe-based enzymatic process. The final libraries were assessed for quality on an Agilent TapeStation (Agilent Technologies, Inc., Wilmington, DE, USA), and quantitative RT-PCR for library quantification was performed using a Kapa Library Quantification Kit (Roche Sequencing, Pleasanton, CA, USA). The libraries were sequenced on two Illumina NextSeq-2000 sequencing runs to generate >90 million pairs of 105-base reads per sample.

### 2.3. Surfaceome Inference

The surfaceome was defined and annotated using data as previously described [14] (R package https://github.com/steveneschrich/surfaceome, accessed on 23 June 2022). Dataset annotations of the surfaceome were performed as follows. Expression data were consolidated to gene symbols (for repeated measurements) using the reporter with the highest median expression and then linking them to SURFY data via the symbol, with the exception of MCC3651 which used ensembl gene annotation. Surfaceome statistics for each experiment were calculated as follows. Genes were rank-ordered for each sample, and then the median rank per gene was calculated across samples (data.table and matrixStats R packages). Likewise, the quantile of each gene was computed per sample and then summarized to the median quantile per gene across samples (ecdf and matrixStats). The median quantile for a gene (across samples) was also categorized into Q1–4 representing the quartile of expression (relative to other genes in the samples). The expressed surfaceome of a dataset consisted of genes in quartile 4 of overall gene expression. We defined the PSCC-expressed surfaceome as the intersection of Q4 expressed surfaceomes from the cell line and MCC3651 datasets.

Surfaceome annotations included (1) Almén main and subclass protein family categorizations [26] and (2) glycosylation motifs (*O*- and *C*-) as well as non-cytosolic *N*-glycosylation (noncyt nxst; [N-X-S/T]) as annotated in GlycoMine [27]. For the glycosylation motif annotations, the data were transformed to having any called sites (glycomineO, glycomineC, noncyt, nxst) present prior to analysis.

### 2.4. Immunohistochemistry

Twelve formalin-fixed, paraffin-embedded (FFPE) PSCC tissue blocks were retrieved and sectioned, and underwent pathologic quality control assessment for the presence of a tumor by an experienced pathologist. Each tumor sample previously underwent HPV assessment and p16 IHC immunostaining as described above. Antibodies for CD147 (Abcam Cat#: ab194401), fibroblast growth factor receptor 1 (FGFR1; Abcam Cat#: ab63601), and monocarboxylate transporter 1 (MCT1/*SLC16A1*; Abcam Cat#: ab85021) were used to assess protein expression in the tissue using standard IHC methods. Staining was classified based on intensity (none (0), weak (1), moderate (2), and strong (3)) and percentage of positively stained tumor cells by localization in the nuclear and plasma membrane compartments with categorizations as less than or equal to 25% (1), 30–60% (2), and greater than or equal to 61% (3).

### 2.5. Statistical Analysis

Statistical analyses were performed using the R statistical software (R4.2.2). Summary tables and crosstabs were generated using gtsummary and flextable. Figures were generated using ComplexHeatmap, ggplot2, ggpubr, patchwork, and ggvenn. HPV differences in MCC3651 were calculated using a *t*-test. Survival plots were generated using survminer and differences in survival were computed using log-rank tests. IHC differences were assessed using boxplots and Wilcoxon statistical tests (ggpubr). Differences in presence of glycosylation motifs were assessed using Pearson’s chi-squared test (gtsummary). Patient characteristics were compared using Wilcoxon or Fisher’s exact tests (see tables for details).

## 3. Results

### 3.1. PSCC Cell Line Surfaceome Characterization

To begin to characterize the surfaceome in PSCC, we first evaluated a previously published translatomic dataset from five patient-derived, HPV-negative (HPV-neg), PSCC cell lines (two lines with epithelial morphology and three lines with cancer- associated fibroblast [CAF] features). The characteristics of the five cell lines from origin tumors are described in Supplemental Table S2 [18]. Of note, epithelial cell2 was derived from a patient with HPV16 infection, but this was lost during subsequent cell passages.

**Figure 1:**
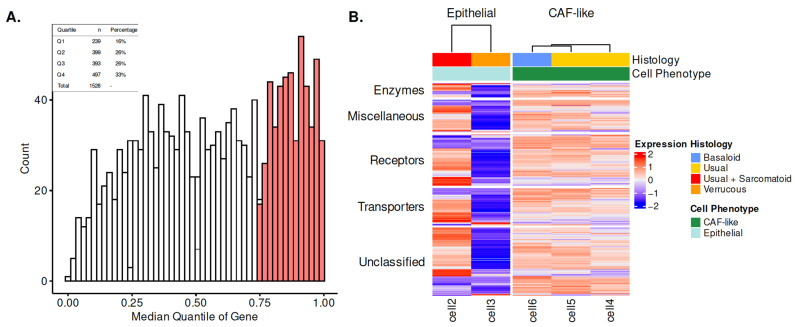
PSCC cell line surfaceome characterization. (**A**) Distribution of surfaceome genes across PSCC cell lines. Top quartile of expression is shaded and is classified as the expressed surfaceome. (**B**) Heatmap of functional protein classes in PSCC cell lines, which are grouped by cell type and tumor origin histology. Abbreviations: CAF, cancer-associated fibroblast.

By starting with ribosome-bound transcript pools, we inferred that the probability of translation to a protein product is increased, which is necessary for surface localization. Beginning with the translatomic data, we evaluated overlap with the curated surfaceome list from the SURFY prediction tool (2886 surfaceome candidates), which resulted in 1528 unique candidates in PSCC cells. As surfaceome genes have variable expression across cell lines (Figure 1A), we hypothesized that concordantly and highly expressed genes (top 25%, shaded bars) represented potential high priority targets.

This approach resulted in 497 genes that were used as the ‘expressed’ PSCC cell line surfaceome (Supplemental Table S3). A heatmap demonstrates the expression levels of these surfaceome genes and their associated functional protein classes defined by Almén et al. [26] in each cell line (Figure 1B). When evaluating the epithelial cell lines, clear differences were noted in the surfaceome composition of verrucous histology. The CAF-like cells demonstrated similar surfaceome expression patterns with each other, despite divergent origin histology, which also overlapped with epithelial cells. Table 1 demonstrates the breakdown of the functional classes within the expressed surfaceome, which identified that 25%, 18%, and 7% of targets were categorized as receptors, transporters, and enzymes, respectively. The breakdown of functional protein subclasses by expression quartiles is shown in Supplemental Table S4.

The SURFY database also includes data derived from GlycoMine [27], a motif analysis tool for the most abundant glycosylation events (e.g., *N*-glycosylation (non- cytoplasmic; *N*-X-S/T), *O*-glycosylation, and *C*-mannosylation). When comparing the bottom and top quartiles of surfaceome gene expression, we found significant differences in the presence of *O*-linked (*p* < 0.001) and *N*-linked (*p* = 0.024) glycosylation motifs. There was no difference in *C*-linked glycosylations between quartiles of expression (Table 2). To our knowledge, this represents the first characterization of the surfaceome in PSCC cell lines as well as an accompanying glycosylation motif inference.

### 3.2. Patient Tumor Surfaceome and Druggability Potential

Next, we evaluated the expressed surfaceome within an independent and ethnically diverse cohort of 18 patient-derived primary PSCC samples profiled by RNA-seq (patient and tumor characteristics shown in Table 3). Of note, 8 PSCCs were HPV-negative (HPV-neg) and 10 were HPV-positive (HPV-pos). All HPV-pos tumors stained positive for p16 (*p* < 0.001).

**Figure 2:**
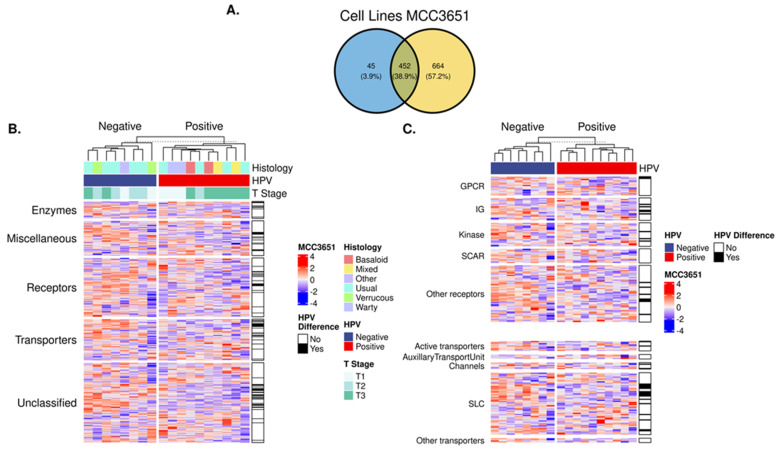
Characterization of patient-derived PSCC surfaceome. (**A**) Venn diagram of cell line (translatomics) and patient tumor (RNA-seq) surfaceome genes (top quartile). (**B**) Heatmap of functional protein classes in PSCC samples grouped by HPV status. (**C**) Heatmap representing receptor and transporter protein subclasses based on HPV status. Significant differences in protein class by HPV status are indicated by black lines in the vertical bar. Abbreviations: GPCR, G-protein-coupled receptor; IG, immunoglobulin superfamily; SCAR, scavenger receptor; SLC, solute carrier.

To begin to describe the surfaceome within patient tumors, we used the expressed cell-line-derived surfaceome profiles to infer expression profiles for tumor cells and potentially CAFs. As expected, surfaceome genes between the cell line translatomic (Clariom^TM^ D platform) and RNA-seq approaches were variable in transcript coverage. The top 25% of expressed surfaceome genes between cell lines and patient tumors (Figure 2A), revealed 452 unique genes (Supplemental Table S5). From this gene set, we inferred the expressed surfaceome profile in PSCCs and their associated functional protein classes (Figure 2B). The most abundant and classified protein families were receptors (n = 115; 25%) and transporters (n = 80; 18%). Notably, 156 (35%) surfaceome targets have no functional classification.

We also observed differences in the expression levels of functional protein classes between HPV-pos and HPV-neg tumors (shown as black lines in the vertical bar adjacent to heatmap). In this regard, 25%, 21%, and 15% of transporters, enzymes, and receptors, respectively, demonstrated differences according to the HPV status (Table 4).

The protein family subclass differences by HPV status are shown in Figure 2C (Supplemental Table S6). Within the transporter class, the largest differences were seen within channels (2/6; 33%) and solute carrier family (SLC) members (16/57; 28%). The most notable differences in the receptor subclass were immunoglobulin superfamily (IgSF) members (5/19; 26%) and G-protein-coupled receptors (GPCR) (2/16; 12%). No differences were found in the proportions of scavenger receptors (0/12; 0%) by HPV status.

To validate our findings, we evaluated the expressed surfaceome in two previously published PSCC patient cohorts with accompanying transcriptome data [23,25]. The platforms used to profile gene expression in these datasets were limited in the breadth of transcriptome coverage compared to our RNA-seq data. Notably, the Johnstone cohort and GSE57955 datasets included 338 and 326 surfaceome genes, respectively. The overlap of the datasets with the expressed surfaceome identified 123 genes, which also showed differences according to the HPV status (Supplemental Figure S1). Overall, these data suggest that most expressed surfaceome genes are independent of HPV infection in PSCC (81% of the surfaceome), but within some protein subclasses, HPV infection may influence the surfaceome profile.

Given the dearth of data describing cell surface accessible targets in PSCC, we evaluated the potential druggability of the expressed surfaceome list by cross-referencing this with drugs annotated in the DrugBank database [28]. Within the annotated surfaceome list, the receptor class had the highest proportion of target–drug hits (n = 27; 23.4%) with 85 potential compounds. Within the transporter class, 18 target–drug hits were identified by 56 compounds. Table 5 summarizes the potential druggable surfaceome classes.

### 3.3. Validation of PSCC Surfaceome Protein Expression

**Figure 3:**
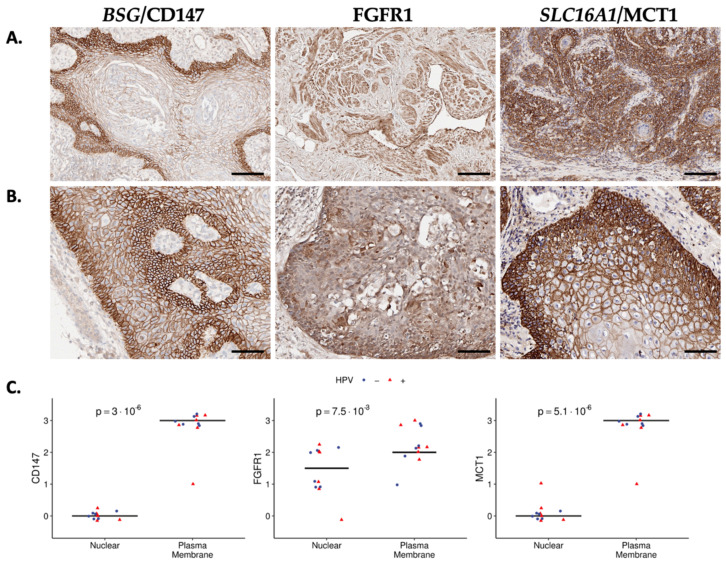
Expression of select surfaceome proteins from top quartile of gene expression. (**A**) Primary PSCCs (n = 12) were stained for BSG/CD147 (**left**), FGFR1 (**middle**), and *SLC16A1*/MCT1 (**right**) proteins; top row demonstrates representative staining patterns. (**B**) Magnification (20×; scale bar is 50 μm) of representative sections demonstrating nuclear vs. membrane localization. (**C**) Percent of cells with protein expression at the plasma membrane vs. the nuclear compartment (see Section 2 for details). Tumor samples were labeled by HPV status (blue circle = HPV-neg; red triangle = HPV-pos).

The above analyses were based on transcript levels, which may not fully recapitulate protein expression due to various post-transcriptional regulation steps, spatiotemporal diversity in expression among individual cells or within tumor compartments, and differences in transcription rates and protein stability [29]. Thus, we evaluated three surfaceome targets identified in the top quartile of expression, which represented different functional protein classes. These selected surfaceome markers were BSG/CD147 (99%; unclassified), FGFR1/FGFR1 (85%, receptor), and SLC16A1/MCT1 (97%; transporter); see Supplemental Table S5. IHC staining in 12 primary PSCC samples (evenly split by HPV status) demonstrated robust expression (2–3+) of each protein at the cell surface (Figure 3A,B) and quantification of surface versus nuclear staining was significant for each marker (Figure 3C). FGFR1 staining demonstrated more diversity in membrane versus nuclear localization compared to CD147 and MCT1, with the latter having a minimal nuclear signal.

### 3.4. Prognostic Association of Select Surfaceome Targets

**Figure 4:**
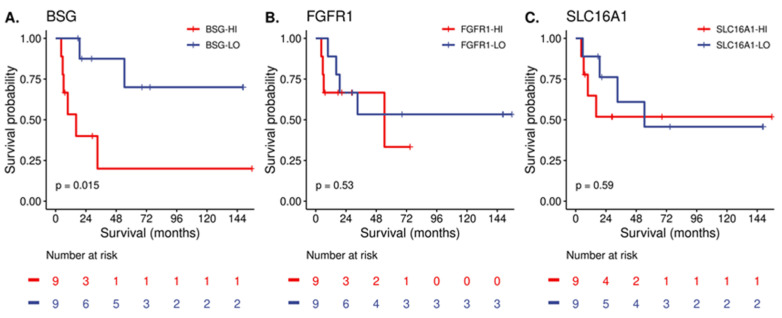
Overall survival association with select surfaceome gene expression. Target genes (**A**) BSG/CD147, (**B**) FGFR1, (**C**) SLC16A1/MCT1 were dichotomized into high and low by the median gene expression value. Kaplan–Meier estimates are shown with log-rank comparison between groups.

Last, we explored whether these highly expressed surfaceome targets may be associated with patient overall survival (OS). The median OS for the cohort was 54.5 months. Of the three selected targets, we found that only PSCCs with elevated expression of *BSG* demonstrated inferior median OS compared to those with low expression (16.2 months versus not reached; *p* = 0.015) (Figure 4).

## 4. Discussion

The cell surface is a prime location for biomarker exploration and therapeutic target discovery in oncology, which is underscored by clinical experience demonstrating that targeting cell-surface-localized proteins with small molecule inhibitors or monoclonal antibodies facilitates tumor regression by various means [30]. Also, interrogating the surfaceome opens additional opportunities to prioritize more advanced therapeutics, such as antibody–drug conjugates (ADCs) or cell-based therapies [31,32,33].

Several computational tools have used motif analysis to infer protein localization at the plasma membrane and subcellular compartments via high throughput methods. Bausch-Fluck et al. complemented these workflows with a chemoproteomic Cell Surface Capture technology and mass spectrometry to validate protein presence at the cell surface [34]. This work provided a public resource, the CSPA [13], which has served as a starting reference for the human cell surfaceome. The CSPA was then used as a validated training set for a machine-learning approach called SURFY, which used domain-specific features to propose an expanded human surfaceome list of 2886 proteins in more than 600 human cell lines [14]. Several investigators have built on SURFY to interrogate the cell surfaceome in cell lines and patient tumors [17,35]. Notably, none of these studies evaluated PSCC; thus, we sought to characterize HPV-independent and HPV-dependent cell surface protein compositions in a similar manner.

We began with translatomic data from patient-derived PSCC cell lines (epithelial and CAF-like), all of which were HPV-negative. These cell line results allowed us to characterize the surfaceome of cells that contributes to tumor progression and treatment resistance [36] independent of heterogeneous tumor cell–cell and cell–matrix architectures. With this approach, we categorized high priority targets, assuming that the top quartile of expression was representative of the most abundant surface proteins. As anticipated, receptors made up about a quarter of the surfaceome followed by transporters (18%) in the cell lines. Representative receptors in the top 90% of the expression distribution included various cytokine receptors, such as *IFNAR2*, *IFNGR1*, *IL13RA1*, and *IL1R1*, which may suggest that PSCC cells are primed to receive external immunomodulatory stimuli. Other potential immunoregulatory proteins include the scavenger receptors (*MRC1, CD302)*, which may facilitate phagocytosis of pathogenic antigens or facilitate cell–cell communication [37,38]. Of interest, the previously described epidermal growth factor receptor (EGFR) was in the top 84% of expression. Transporters in the top 90% of expression included various amino acid transporters (e.g., *SLC1A5*, *SLC38A1, SLC7A5*). *SLC7A5* (LAT1) has been implicated in various cancers and may facilitate treatment resistance by modulating mTORC1 signaling [39], although its role in PSCC is unknown.

Glycosylation is a major post-translational modification and is responsible for decorating various protein domains, including those facing the extracellular milieu. Given the importance of glycosylation in regulating various aspects of surfaceome composition, organization, and stabilization [40], we evaluated this further in the PSCC cell lines. Interestingly, surfaceome genes within the top quartile of expression had approximately double the proportion of *O*-glycan motifs compared to those in the lowest quartile. This may reflect prior data suggesting at least 80% of proteins undergoing transit through the secretory compartment and/or localization to the surface undergo this modification [41]. Prior evidence has found that HPV oncogenic E6/E7 proteins influence aberrant glycosylation patterns in cervical cancer [42,43], which may also potentially modulate the immune–tumor interface [44]. Similarly, HPV has been shown to influence the *N*-glycome in head and neck cancer cells [45]. Whether HPV influences the PSCC glycome is unknown, but may represent a novel area of investigation to understand tumor–immune cell interactions, especially given that PSCC tends to have a lower mutation burden [46] and lower response rate to immune checkpoint blockade therapy in non-selected patients [47].

The HPV oncogenic proteins E6/E7 contribute to PSCC biology in various ways by altering p53 activity, relaxing cell cycle checkpoints, instigating genomic instability, and altering DNA damage repair capacity [48,49]. Among this HPV-altered cellular state, various receptors and their downstream effectors may also be aberrantly activated, such as the EGFR and PI3K/Akt, mTOR, or JAK/STAT signaling pathways [50]. Many of these HPV-related events have also been described in PSCC [19,25,51,52], although there is currently little data on HPV-related differences in the surfaceome. Our data suggest that the majority of surfaceome constituents (81%) in patient tumors are independent of HPV infection. This may imply that various surfaceome proteins are more related to the penile origin and could represent an opportunity for pan-PSCC drug development efforts. Also, we identified heterogeneity in surfaceome expression within the broad groupings of HPV infection status. This expression diversity may be driven by variation in the high-risk HPV type or host response [53], which may act as contributors to surfaceome gene expression. This requires further future investigation to understand this impact in PSCC.

Although the expression of most surfaceome genes appears independent of HPV, we did observe that about a quarter of transporters varied in expression. Specifically, the SLC transporter family demonstrated almost 30% differential expression based on HPV, which was primarily represented by amino acid transporters. These included the heterodimeric amino acid heavy chain *SLC3A2*/4F2 and catalytic light chain subunits *SLC7A5/6* (LAT1/2) [54] as well as the lactate transporter *SLC16A1*/MCT1 [55]. Intriguingly, the expression of various metabolic transporters appear to be different based on HPV status, which may reflect the biomass needed to sustain viral-mediated oncogenic events. In this regard, our IHC analysis demonstrated that MCT1 is robustly expressed in PSCC and is localized to the plasma membrane. Notably, *BSG* was found to be prognostic of OS in our patient cohort (Figure 4). *BSG*/CD147 (also called EMMPRIN) is an IgSF multi-functional protein that may play a role in the metabolic phenotype of cells by regulating glucose and lactate transport [56], fatty acid metabolism [57], and amino acid transport [58]. Several studies have described a role of CD147 in various cancers, which is supported by a meta-analysis that found that CD147 is associated with adverse clinical outcomes and may represent a potential therapeutic target [59]. In this regard, a radiolabeled monoclonal antibody targeting CD147 was evaluated in a randomized, phase 2, clinical trial in hepatocellular carcinoma patients and found that targeting CD147 doubled tumor control (43% vs. 22%) [60]. Our IHC analysis also demonstrated that CD147 is strongly expressed in PSCC and at the plasma membrane, which could represent a potential biomarker or target for this malignancy, although this requires further investigation. Future research is also needed to define the metabolic milieu of PSCC and evaluate if these features represent vulnerabilities for targeting.

Several limitations should be noted in the present study. First, our initial categorization of the surfaceome was dependent on ribosome-bound transcripts in HPV-negative and heterogenous patient-derived cell lines versus single-cell transcriptomics from patient tumors. Although starting with cell lines removes the cell–cell context in tumors, which likely influences gene expression patterns in the surfaceome, the strength of starting with translatomic data provides an opportunity to isolate actively translating mRNAs that have a high probability of becoming surface proteins. Also, starting with HPV-negative cells provided the ability to examine the pan-PSCC surfaceome, which may cooperate with subsequent HPV infection during carcinogenesis if the basal surfaceome state is influenced more by cell origin versus viral infection. Even though we used the top quartile of expression to describe the expressed surfaceome, it is possible that other proteins with lower expression are targetable. For example, we previously identified Nectin-4 (not in top 25% of surfaceome distribution) as a potential target in PSCC, which may be targeted by the FDA-approved ADC enfortumab vedotin [20]. Lastly, the starting surfaceome list was based off in silico inferences rather than direct measurement at the cell surface. While we did not individually validate the hundreds of surfaceome proteins identified, our protein expression analysis confirmed that targets at the higher end of the expression distribution were localized to the cell surface compared to other subcellular compartments. Overall, this work provides the first step in describing the PSCC surfaceome and may serve as a valuable tool for rare genitourinary cancer researchers.

## 5. Conclusions

In conclusion, this work provides the first description of the surfaceome in PSCC cell lines and patient tumors, while also providing a snapshot of potential surfaceome differences by HPV status. Due to the rarity of PSCC and lack of available translational research resources, patients with PSCC have not been afforded access to novel treatment approaches compared to patients with more common tumor types. Thus, profiling the surfaceome can aid in prioritizing cell surface targets for future biomarker and drug targeting strategies.

## Figures and Tables

**Table 1:** Functional protein group breakdown of the PSCC cell line surfaceome.

Characteristic	N = 497 ^1^
Almén Category	
Enzymes	35 (7%)
Miscellaneous	79 (16%)
Receptors	125 (25%)
Transporters	91 (18%)
Unclassified	167 (34%)

^1^ n (%).

**Table 2:** Comparison of glycosylation motifs by quartiles of expression in PSCC cell lines.

Characteristic	Q1, N = 239 ^1^	Q4, N = 497 ^1^	*p*-Value ^2^
glycomineO_present	24 (10%)	111 (22%)	<0.001
glycomineC_present	45 (19%)	97 (20%)	0.5
noncyt. nxst present	224 (94%)	483 (97%)	0.024

^1^ n (%); ^2^ Pearson’s Chi-squared test.

**Table 3:** Clinicopathologic characteristics of the MCC3651 cohort.

Variable	HPV Negative n = 8	HPV Positive n = 10	*p*-Value
Tissue Source			
Penis	8 (100%)	10 (100%)	
Age at Surgery	58 (50, 67)	58 (52, 65)	>0.9
Race			0.11
Asian	2 (25%)	0 (0%)	
Black	0 (0%)	2 (20%)	
Hispanic	1 (12%)	0 (0%)	
White	5 (62%)	8 (80%)	
Histology			0.086
Basaloid	0 (0%)	2 (20%)	
Mixed	0 (0%)	2 (20%)	
Other	1 (12%)	0 (0%)	
Usual	5 (62%)	4 (40%)	
Verrucous	2 (25%)	0 (0%)	
Warty	0 (0%)	2 (20%)	
LVI			0.6
No	3 (38%)	2 (20%)	
Yes	5 (62%)	8 (80%)	
p16 IHC			<0.001
Negative	6 (75%)	0 (0%)	
Positive	1 (12%)	10 (100%)	
Unknown	1 (12%)	0 (0%)	
pT			0.2
1	2 (25%)	3 (30%)	
2	4 (50%)	1 (10%)	
3	2 (25%)	6 (60%)	
pN			0.6
0	3 (38%)	1 (10%)	
1	0 (0%)	1 (10%)	
2	4 (50%)	5 (50%)	
3	1 (12%)	3 (30%)	
n (%); median (IQR).			
Wilcoxon rank sum exact test; Fisher’s exact test.			

**Table 4:** Functional protein group differences by HPV status in patient tumors.

Almén Category	MCC 3651 (% Difference by HPV Status)
Enzymes	7/33 (21%)
Miscellaneous	13/68 (19%)
Receptors	17/115 (15%)
Transporters	20/80 (25%)
Unclassified	29/156 (19%)

**Table 5:** Evaluation of the potential druggability of the surfaceome.

Almén Category	N	Total # Drugs	Mean # Drugs	Targets with Drug(s)	Percent Targets with Drug(s)
Receptors	115	85	0.74	27	23.47
Transporters	80	56	0.70	18	22.50
Unclassified	222	25	0.11	14	6.31
Enzymes	33	12	0.36	7	21.21
Miscellaneous	69	1	0.01	1	1.45

## Data Availability

The data presented in this study are available in this article.

## Supplementary Materials

The following supporting information can be downloaded at: https://www.mdpi.com/article/10.3390/cancers15143636/s1, Figure S1: External datasets employed for validation of the PSCC surfaceome; Table S1: Analyzed Datasets; Table S2: Cohort Characteristics of PSCC Cell Line Origins; Table S3: Expressed Surfaceome in PSCC Cell Lines (n = 497); Table S4: Protein Family Subclasses by Expression Quartile in PSCC Cell Lines; Table S5: Expressed Surfaceome in PSCC Tumors (n = 452); Table S6: Protein Family Subclasses by HPV Differences in PSCC Tumors.
