# Supplementary figures and images for: An Assessment of the Penile Squamous Cell Carcinoma Surfaceome for Biomarker and Therapeutic Target Discovery
Source: Cancers (Basel). 2023 Jul 15;15(14):3636. doi: 10.3390/cancers15143636 (PMC10377392; doi:10.3390/cancers15143636)

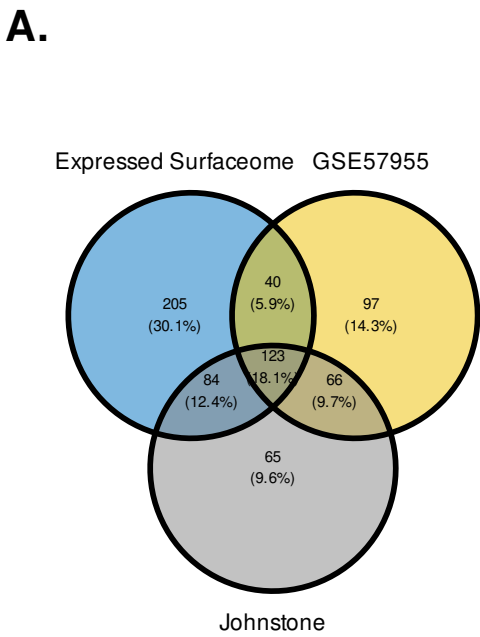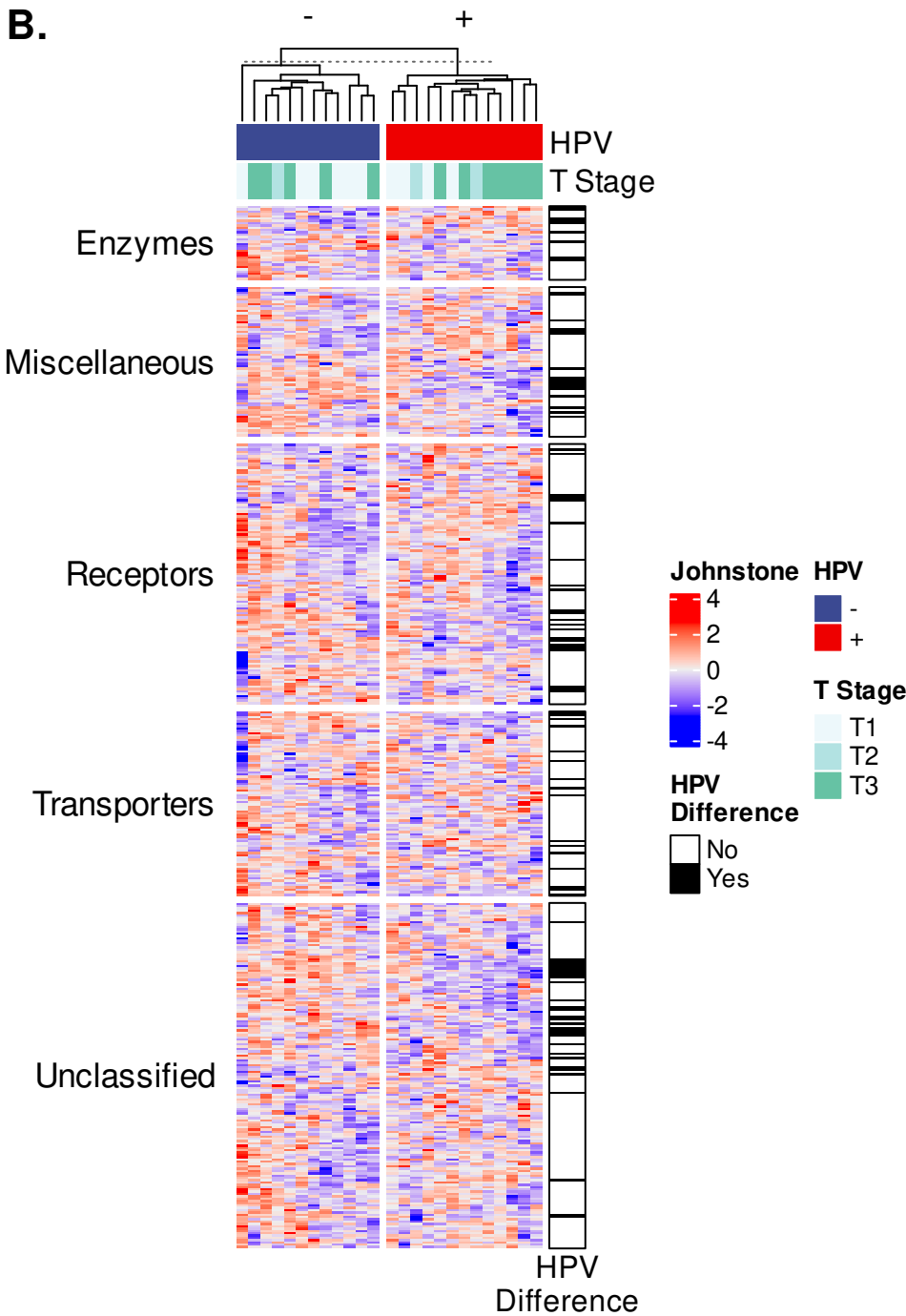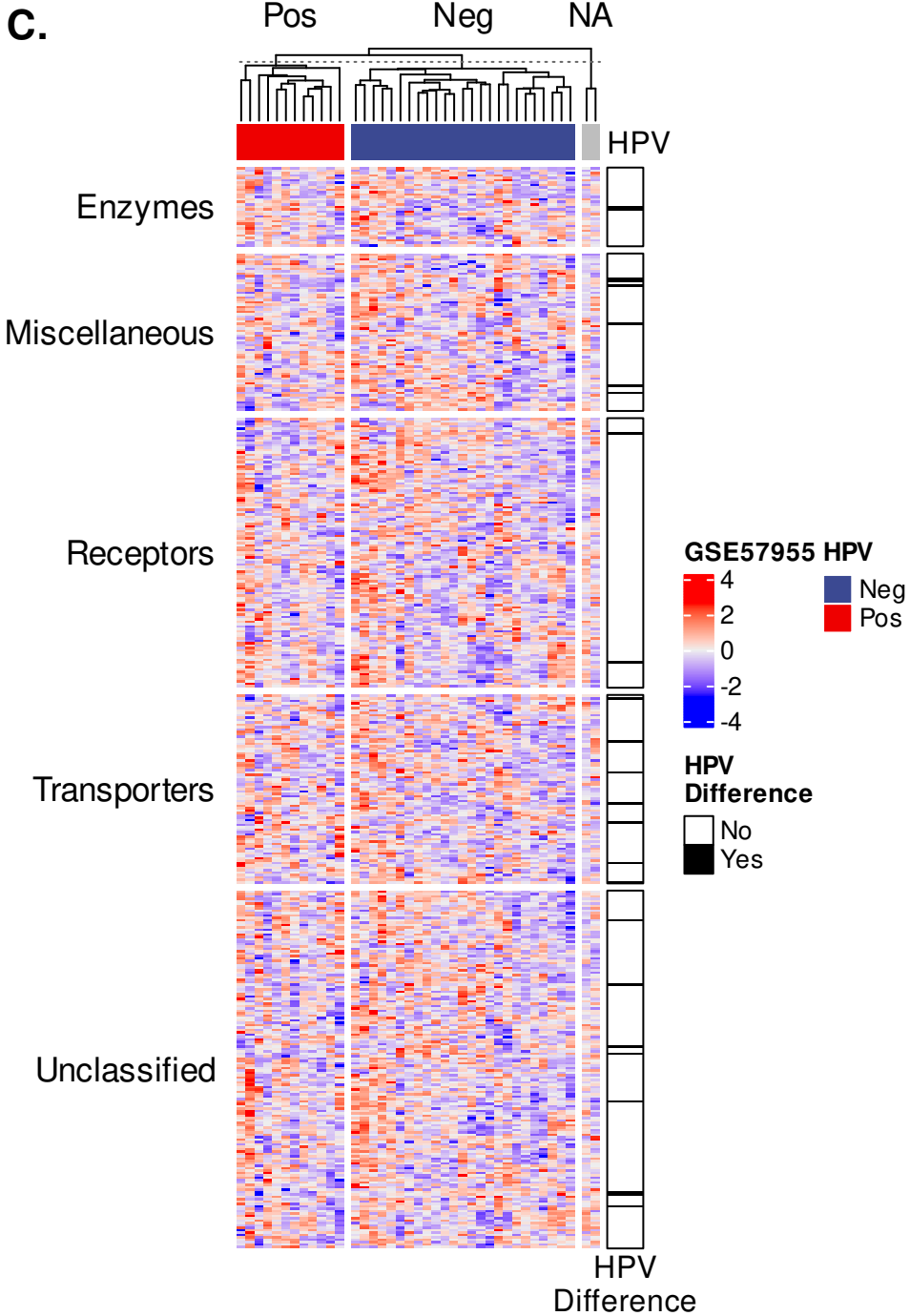

**D.**

| Almen category | GSE57955   | Johnstone    |
|----------------|------------|--------------|
| Enzymes        | 2/33 (6%)  | 9/32 (28%)   |
| Miscellaneous  | 6/66 (9%)  | 17/65 (26%)  |
| Receptors      | 2/113 (2%) | 22/113 (19%) |
| Transporters   | 7/79 (9%)  | 16/80 (20%)  |
| Unclassified   | 8/150 (5%) | 31/150 (21%) |

Supplement: Supplementary file 1 [file cancers-15-03636-s001.zip › cancers-2455968-supplementary figure.pdf]
